# Supplementary material for: Practitioner’s perspectives on access to Rabies Post-Exposure Prophylaxis in Tanzania: A mixed-methods and theoretically-informed study to inform policy and practice
Source: PLOS Glob Public Health. 2026 May 14;6(5):e0005595. doi: 10.1371/journal.pgph.0005595 (PMC13175354; doi:10.1371/journal.pgph.0005595)
Supplement: Attachment — Submitted filename: Response to reviewers.docx [file pgph.0005595.s003.docx]

**Response to the reviewer**

**Journal Requirements:**

1. Please amend your detailed Financial Disclosure statement. This is published with the article. It must therefore be completed in full sentences and contain the exact wording you wish to be published.

i. Please clarify all sources of financial support for your study. List the grants, grant numbers, and organizations that funded your study, including funding received from your institution. Please note that suppliers of material support, including research materials, should be recognized in the Acknowledgements section rather than in the Financial Disclosure. 
ii. State the initials, alongside each funding source, of each author to receive each grant. For example: "This work was supported by the National Institutes of Health (####### to AM; ###### to CJ) and the National Science Foundation (###### to AM)."
iii. State what role the funders took in the study. If the funders had no role in your study, please state: “The funders had no role in study design, data collection and analysis, decision to publish, or preparation of the manuscript.”
iv. If any authors received a salary from any of your funders, please state which authors and which funders.

*We have now revised the Financial Disclosure statement to comply with the journal’s requirements.*

2. We ask that a manuscript source file is provided at Revision. Please upload your manuscript file as a .doc, .docx, .rtf or .tex.

*The revised manuscript source file has now been uploaded in .docx format.*

3. Please ensure that the Title in your manuscript file and the Title provided in your online submission form are the same.

*The manuscript title has now been corrected so that the title in the manuscript file and in the online submission system are identical. The revised title now reads: “Practitioners’ perspectives on access to Rabies Post-Exposure Prophylaxis in Tanzania: A mixed-methods and theoretically informed study to inform policy and practice.”*

4. Thank you for uploading your study's underlying data set. Unfortunately, the repository you have noted in your Data Availability statement does not qualify as an acceptable data repository according to PLOS's standards. At this time, please upload the minimal data set necessary to replicate your study's findings to a stable, public repository (such as figshare or Dryad) and provide us with the relevant URLs, DOIs, or accession numbers that may be used to access these data. For a list of recommended repositories and additional information on PLOS standards for data deposition, please see <https://journals.plos.org/plosone/s/recommended-repositories>.

*We apologise for this oversight. We have now deposited the minimal data set and associated analysis code necessary to replicate the study’s findings in a stable, public repository that meets PLOS standards (Zenodo). We updated the Data Availability statement accordingly and include the relevant DOI and access link: (https://zenodo.org/records/18508654).*

Some material included in your submission may be copyrighted. According to PLOS’s copyright policy, authors who use figures or other material (e.g., graphics, clipart, maps) from another author or copyright holder must demonstrate or obtain permission to publish this material under the Creative Commons Attribution 4.0 International (CC BY 4.0) License used by PLOS journals. Please closely review the details of PLOS’s copyright requirements here: PLOS Licenses and Copyright. If you need to request permissions from a copyright holder, you may use PLOS's Copyright Content Permission form.

Please respond directly to this email or email the journal office and provide any known details concerning your material's license terms and permissions required for reuse, even if you have not yet obtained copyright permissions or are unsure of your material's copyright compatibility.

Potential Copyright Issues:

a. Figures 1 and 3: please (a) provide a direct link to the base layer of the map (i.e., the country or region border shape) and ensure this is also included in the figure legend; and (b) provide a link to the terms of use / license information for the base layer image or shapefile. We cannot publish proprietary or copyrighted maps (e.g. Google Maps, Mapquest) and the terms of use for your map base layer must be compatible with our CC-BY 4.0 license.

Note: if you created the map in a software program like R or ArcGIS, please locate and indicate the source of the basemap shapefile onto which data has been plotted.

If your map was obtained from a copyrighted source please amend the figure so that the base map used is from an openly available source. Alternatively, please provide explicit written permission from the copyright holder granting you the right to publish the material under our CC-BY 4.0 license.

Please note that the following CC BY licenses are compatible with PLOS license: CC BY 4.0, CC BY 2.0 and CC BY 3.0, meanwhile such licenses as CC BY-ND 3.0 and others are not compatible due to additional restrictions.

If you are unsure whether you can use a map or not, please do reach out and we will be able to help you. The following websites are good examples of where you can source open access or public domain maps:

* U.S. Geological Survey (USGS) - All maps are in the public domain. ([http://www.usgs.gov](http://www.usgs.gov/)) 
* PlaniGlobe - All maps are published under a Creative Commons license so please cite “PlaniGlobe, [http://www.planiglobe.com](http://www.planiglobe.com/), CC BY 2.0” in the image credit after the caption. (<http://www.planiglobe.com/?lang=enl>) 
* Natural Earth - All maps are public domain. (<http://www.naturalearthdata.com/about/terms-of-use/>)

If the reviewer comments include a recommendation to cite specific previously published works, please review and evaluate these publications to determine whether they are relevant and should be cited. There is no requirement to cite these works unless the editor has indicated otherwise.

*Figures 1 and 3 in this manuscript were created by the authors and were not copied from third-party or proprietary sources. These figures were generated using open-access shapefiles for Tanzania. Similarities between Figures 1 amd 3 in the current manuscript and Figure 2 in* [*Lushasi et al., 2020*](https://www.frontiersin.org/journals/public-health/articles/10.3389/fpubh.2020.00013/full) *(Frontiers in Public Health) arises because:*

- *The studies were conducted in the same geographical regions of Tanzania,*
- *The work was implemented by the same research team, and*
- *The maps were generated using the same openly available shapefiles.*

*Importantly, this does not constitute reuse of copyrighted third-party material. The maps were independently generated for each manuscript, and although they use the same base shapefiles, they convey different analytical content and messages relevant to the respective studies. The lead author of both publications is the same, and the senior authorship also overlaps, reflecting continuity of the research programme rather than reuse of external material.*

*The base map shapefiles used to generate Figures 1 and 3 are derived from open-access sources compatible with CC BY 4.0, and are not proprietary (e.g., Google Maps or Mapquest). The exact shapefiles used to generate these figures have now been included in the reproducible data repository associated with this manuscript, and made publicly available via Zenodo: https://zenodo.org/records/18508654.*

*No additional copyright permissions are required, as all map components are either author-generated or derived from openly licensed/public-domain sources that are fully compatible with the PLOS CC BY 4.0 license.*

*We trust this clarification resolves the concern, and we are happy to provide any further details if required.*

**Reviewers' comments:**

5. Does this manuscript meet PLOS Global Public Health’s [publication criteria](https://track.editorialmanager.com/CL0/https:%2F%2Fjournals.plos.org%2Fglobalpublichealth%2Fs%2Fcriteria-for-publication/1/010f019bb0e58528-4186bffa-e100-4abc-a4f8-6566e0e682d5-000000/qyk4gTpMmF5IuCfjpdXfRr4ngaQgl6XWkae0ka8EOUc=245)? Is the manuscript technically sound, and do the data support the conclusions? The manuscript must describe methodologically and ethically rigorous research with conclusions that are appropriately drawn based on the data presented.

Reviewer #1: Yes; Reviewer #2: Yes: Reviewer #3: Yes

6. Has the statistical analysis been performed appropriately and rigorously?

Reviewer #1: I don't know; Reviewer #2: Yes and Reviewer #3: N/A

7. Have the authors made all data underlying the findings in their manuscript fully available (please refer to the Data Availability Statement at the start of the manuscript PDF file)?
   The [PLOS Data policy](https://track.editorialmanager.com/CL0/https:%2F%2Fjournals.plos.org%2Fglobalpublichealth%2Fs%2Fdata-availability/1/010f019bb0e58528-4186bffa-e100-4abc-a4f8-6566e0e682d5-000000/DQsFecuCfny7w9MvqyBmUbW_hNJR45ZMVnAw4vSXNjM=245) requires authors to make all data underlying the findings described in their manuscript fully available without restriction, with rare exception. The data should be provided as part of the manuscript or its supporting information, or deposited to a public repository. For example, in addition to summary statistics, the data points behind means, medians and variance measures should be available. If there are restrictions on publicly sharing data—e.g. participant privacy or use of data from a third party—those must be specified.

Reviewer #1: Yes; Reviewer #2: Yes ;Reviewer #3: Yes

8. Is the manuscript presented in an intelligible fashion and written in standard English?
   PLOS Global Public Health does not copyedit accepted manuscripts, so the language in submitted articles must be clear, correct, and unambiguous. Any typographical or grammatical errors should be corrected at revision, so please note any specific errors here.

Reviewer #1: Yes; Reviewer #2: Yes Reviewer #3: Yes

*We sincerely thank the reviewers for their time and for confirming that the manuscript meets the journal’s publication criteria and that the data support the conclusions and note that no concerns were raised about the statistical analyses. The Data Availability Statement has been updated, and the DOI for the Zenodo repository hosting the data and analysis code has been added to facilitate open and unrestricted access. We have conducted a final language review and addressed any minor typographical or grammatical issues in the revised version*

9. Review Comments to the Author
   Please use the space provided to explain your answers to the questions above. You may also include additional comments for the author, including concerns about dual publication, research ethics, or publication ethics. (Please upload your review as an attachment if it exceeds 20,000 characters)

**Reviewer #1**: The authors have done appreciable work in collecting data on the health care issues related to Rabies prevention in Tanzania. I have the following observations. Some more statistics ie tables etc may be added to the article to justify the results and discussion. The tables should then be discussed in detail with a conclusion pointing out the steps needed to be taken to improve the prevalent chaos in their health care system.

*Thank you for this constructive feedback. We have updated the conclusion to provide a detailed set of tabulated recommendations (refer to line 510), to strengthen access to PEP in this setting, mapping these to our framework constructs. We have also tried to make our results clearer with improved cross referencing to the discussion, and with additional changes guided by the feedback from the other reviewers.*

Costs per patient could be estimated, inclusive of loss of wages etc. this will further improve the impact of the article.

*Thank you for this thoughtful comment. Our analysis was conducted from the perspective of frontline health and veterinary workers directly involved in rabies control activities. Accordingly, the data presented reflect the views and experiences of these providers, who manage animal bite patients and conduct animal investigations across the study sites. Unfortunately we did not collect data directly from patients, nor did we estimate patient-incurred costs (such as loss of wages), as these were outside the scope of our provider-focused analysis. We agree that societal and patient-level costs represent important barriers to accessing PEP and contribute substantially to the overall disease burden. We have therefore acknowledged this as a limitation of our study in the Discussion section (see lines 470-472).*

I couldn't open the supplementary GITHUB site for more results as it seemed to be a paid site.

*Apologies that access to the supplementary GitHub repository was not clear in the original submission. I have made the linked Github repository public (apologies I had forgotten to do this on the original submission). The Data Availability Statement has now also been updated to include a DOI for a Zenodo repository that permanently hosts the full dataset and analysis code ensuring open and unrestricted access (see line 514).*

**Reviewer #2:**

10. Review of the article "Access to Rabies Post-Exposure Prophylaxis in Tanzania: A Mixed-Methods and Theoretically Informed Study to Inform Policy and Practice"
    This article is a comprehensive study addressing a critical global public health issue: access to rabies post-exposure prophylaxis (PEP) in Tanzania. The study adheres strictly to scientific research principles and is highly relevant, clearly structured, and methodologically rigorous. The use of mixed methods (quantitative analysis of large-scale data from the Integrated Bite Case Management (IBCM) platform and qualitative analysis of healthcare worker communications) within a theoretical model of access to healthcare is a key strength of the study, allowing us not only to document the facts but also to understand the mechanisms and context of barriers. This article makes a significant contribution to both the academic understanding of health systems issues in low- and middle-income countries and to intervention planning, particularly in light of the launch of funding for PEP programs by GAVI (the Vaccine Alliance).

The relevance of this study is undeniable. Rabies remains a deadly, yet 100% preventable, disease that claims tens of thousands of lives annually, primarily among the poorest rural populations in Africa and Asia. The announcement of the "Zero by 30" goal and GAVI's decision to support the procurement of rabies vaccines create a historic window of opportunity. At this moment, field-based evidence is crucial, demonstrating that the mere availability of vaccines in a country is insufficient. This study brings this issue to the forefront by systematically identifying a cascade of interconnected barriers.

The scientific novelty lies in the following:

Application and development of the theoretical model. The authors not only utilize Penchansky and Thomas's classic model (five dimensions of access), but also empirically substantiate and add a sixth dimension—"Appropriateness." This crucial conceptual addition highlights provider competence as a separate, critical access factor, as clearly demonstrated by data on the inappropriate use of RIG or tetanus toxoid.

Depth and Richness of Data. The combination of data on over 10,000 patients with an analysis of real-life healthcare worker conversations and concerns (>3,000 messages) provides a unique, comprehensive picture of the situation. This allows us to move beyond dry statistics ("5% experienced interruptions") to understanding the human tragedies and professional dilemmas behind these figures.

Documenting Emerging Threats. The study documents the emergence of a new, dangerous practice—the use of rabies immunoglobulin (RIG, Equirab) as a substitute for vaccination, which has resulted in fatalities. This is a crucial observation for surveillance systems and regulatory agencies.

The study's methodological framework is exemplary for public health research.

Design: Mixed methods are entirely justified for studying the complex phenomenon of access. The quantitative component objectively captures the scale of the problem (distances, completion rates, interruptions), while the qualitative component reveals the causes, context, and subjective experiences.

Theoretical Framework: The use of a structured conceptual framework (expanded healthcare access framework) lends systematicity to the analysis, prevents fragmented findings, and allows for the classification of barriers into clear categories, which is useful for developing targeted interventions.

Triangulation: The authors consciously employ triangulation, systematically comparing findings from quantitative and qualitative data. This enhances the validity and reliability of the results, allowing for identification of areas where the data complement or corroborate each other.

Ethics: The study has all necessary ethical approvals, and the process for obtaining informed consent from participants (healthcare workers) is described.

Despite its undeniable strengths, the study has a number of limitations, some of which the authors honestly and thoroughly describe in the relevant section. This enhances the credibility of the study.

The absence of a patient voice. This is the main methodological limitation, noted by the authors themselves. The study brilliantly analyzes the system from the service provider's perspective, but the "measure" of acceptability—that is, how much patients trust the service, how comfortable they are, and whether the services meet their cultural expectations—remains unexplored. This is an important gap, as cultural beliefs and stigma can be a significant barrier.

Sample bias. The data only cover those who reached official medical facilities included in the KUSU. The most vulnerable groups—those who immediately turned to traditional healers—didn't have the means to even pay for the first visit.

*We sincerely thank the reviewer for this thoughtful, detailed, and highly positive assessment of our manuscript, and for recognising its relevance, methodological rigor, and contribution to policy and practice. We particularly appreciate the reviewer’s careful reflection on the study’s limitations, including the important observation regarding the absence of a direct patient voice.*

*In response, and to better reflect the lived experiences of those affected by rabies exposure, we have revised the manuscript title to “* ***Practitioner’s perspectives on access to Rabies Post-Exposure Prophylaxis in Tanzania: A mixed-methods and theoretically-informed study to inform policy and practise*** *”. This change was made intentionally to foreground patient experience and ensure that the patient voice and realities of access to care are explicitly captured from the outset of the manuscript, while remaining transparent about the study’s methodological scope.*

**Reviewer #3:**

11. The authors employed a mixed-methods approach, integrating quantitative data from the Integrated Bite Case Management platform with qualitative data from hotline calls and peer-support chats among health and veterinary workers, to identify barriers and facilitators affecting bite victims’ access to post-exposure prophylaxis across four regions of Tanzania. This work addresses a critical and timely public health issue, as inadequate knowledge of post-bite treatment and constraints in access to rabies biologics remain major contributors to preventable rabies deaths in dog-endemic regions, including Tanzania.

I have just one major concern with the manuscript in its current version. While the authors correctly explain early in the Introduction (line 69 and Fig. 2) that post-exposure prophylaxis (PEP) comprises of immediate wound washing, a series of rabies vaccinations, and administration of rabies immunoglobulin (RIG) for previously unvaccinated patients with severe (Category III) exposures, this framework is not consistently or adequately reflected in the subsequent analysis and interpretation. Specifically, across four of the five dimensions of the expanded healthcare access framework, the discussion focuses exclusively on rabies vaccines, with little to no consideration of rabies immunoglobulin (RIG). For patients with severe (Category III) exposures, RIG is arguably the most critical component of PEP, and its omission substantially weakens the analysis. Issues related to availability, accessibility, and affordability should explicitly address RIG, as barriers to RIG access often differ from—and are more severe than—those associated with vaccines. Without clearly incorporating RIG into these dimensions, the manuscript provides an incomplete assessment of the factors influencing access to life-saving PEP. I understand the challenges surrounding RIG access, particularly as the authors allude to in line 363, where they note that the purchase of RIG had not previously been seen in Tanzania. Given this context, the implications of RIG unavailability should be discussed explicitly and in greater depth in the Discussion section. Doing so would help clarify how historical absence, procurement barriers, cost, cold-chain requirements, and limited clinician familiarity contribute to ongoing and future challenges in delivering complete PEP. A clearer, more focused discussion of RIG would strengthen the manuscript by providing a more realistic and policy-relevant assessment of barriers to comprehensive rabies prevention in Tanzania.

*Response*

*Thank you for this important comment. We agree that rabies immunoglobulin (RIG) is often considered to be important for patients with severe (Category III) exposures. However, the limited emphasis in our manuscript on RIG across several access dimensions reflects the historical and practical realities of PEP delivery in Tanzania rather than an omission in our analysis. As shown in the Results, RIG had not previously been procured or used within the public health system within the country, and health workers were only familiar with human rabies vaccines. Thus RIG access was effectively nonexistent at the time our study began.*

*In previous work, we have examined rabies deaths in Tanzania to identify what proportion could have perhaps been prevented with timely vaccine and RIG (Changalucha et et al. 2018, WHO rabies modelling Consortium 2019). In these studies we find negligible benefit to RIG - over 99% of rabies deaths in Tanzania are amongst bite victims who do not initiate timely vaccination. Had they received vaccine, we expect that this would have prevented their deaths.*

*However, use of RIG has been common practice in much of Asia since the 1990s when vaccine quality was poorer. Hence perspectives on the value of RIG reflect its important benefits at this time. Arguably the benefits of RIG today are much less. For these reasons RIG is not the focus of Gavi’s investment in rabies, instead vaccines are. Nonetheless, in countries already using RIG, its use remains high.*

*Critically though, we observe a serious threat if RIG is used instead of vaccine which is what happened in Tanzania recently. RIG began to appear sporadically from late 2022 following marketing tours by private pharmaceutical suppliers. But in the absence of training or guidance, this led to confusion regarding its appropriate use. As documented in our Results, RIG was sometimes incorrectly administered or substituted for rabies vaccine rather than used as an adjunct, with fatal consequences.*

*Because of this historical absence, our data do not meaningfully capture RIG within other access dimensions such as availability, accessibility, or affordability. Health workers did not report RIG-related shortages or cost barriers, largely because it was not part of routine PEP provision during most of the study period. Consequently, we did not have empirical evidence to include RIG under these dimensions in the Results.*

*We have therefore expanded the Discussion to explicitly address the implications of RIG’s historical absence, its recent and poorly regulated introduction, and limited clinician familiarity, while emphasizing its critical role in complete PEP for eligible patients. We believe this provides a clearer and contextually grounded assessment of barriers to comprehensive rabies prevention in Tanzania.*

*Changalucha J, et al (2019). The need to improve access to rabies post-exposure vaccines: Lessons from Tanzania. Vaccine. 2019 Oct 3;37 A45-A53. doi:10.1016/j.vaccine.2018.08.086.*

*WHO Rabies Modelling Consortium (2019). The potential effect of improved provision of rabies post-exposure prophylaxis in Gavi-eligible countries: a modelling study. Lancet Infect Dis.19(1):102-111. doi: 10.1016/S1473-3099(18)30512-7.*
