# Supplementary material for: Practitioner’s perspectives on access to Rabies Post-Exposure Prophylaxis in Tanzania: A mixed-methods and theoretically-informed study to inform policy and practice
Source: PLOS Glob Public Health. 2026 May 14;6(5):e0005595. doi: 10.1371/journal.pgph.0005595 (PMC13175354; doi:10.1371/journal.pgph.0005595)
Supplement: Attachment — Submitted filename: minor revivson.docx [file pgph.0005595.s002.docx]

This article's conclusions and table discuss the need to decentralize PEP services to priority facilities.

I suggest the authors provide more specific criteria or models. For example: "In each ward with a population greater than X and/or located more than Y km from an existing PEP site, at least one medical facility (dispensary or health center) should be designated for rabies storage and vaccination. The selection of these facilities should be based on accessibility to the public (24/7) and the availability of trained personnel." This transforms a general recommendation into a measurable standard.
