# Supplementary material for: Practitioner’s perspectives on access to Rabies Post-Exposure Prophylaxis in Tanzania: A mixed-methods and theoretically-informed study to inform policy and practice
Source: PLOS Glob Public Health. 2026 May 14;6(5):e0005595. doi: 10.1371/journal.pgph.0005595 (PMC13175354; doi:10.1371/journal.pgph.0005595)
Supplement: Attachment — Submitted filename: Response to reviewers_March23.docx [file pgph.0005595.s004.docx]

**Response to the reviewer**

**Reviewer’s comments**

1. If the authors have adequately addressed your comments raised in a previous round of review and you feel that this manuscript is now acceptable for publication, you may indicate that here to bypass the “Comments to the Author” section, enter your conflict of interest statement in the “Confidential to Editor” section, and submit your "Accept" recommendation.Reviewer #1: All comments have been addressed. Reviewer #2: All comments have been addressed. Reviewer #3: All comments have been addressed

2. Does this manuscript meet PLOS Global Public Health’s [publication criteria](https://track.editorialmanager.com/CL0/https:%2F%2Fjournals.plos.org%2Fglobalpublichealth%2Fs%2Fcriteria-for-publication/1/010f019cad98b43b-a982877a-bc1a-4592-b4b0-a8f0b31cb2b3-000000/gsU7w2Y3hX4CQSU64iTt-dZdIfC7j2epbYDLSPxKlDs=252)? Is the manuscript technically sound, and do the data support the conclusions? The manuscript must describe methodologically and ethically rigorous research with conclusions that are appropriately drawn based on the data presented. Reviewer #1: Yes. Reviewer #2: Yes . Reviewer #3: Yes

3. Has the statistical analysis been performed appropriately and rigorously? Reviewer #1: Yes. Reviewer #2: Yes; Reviewer #3: N/A

4. Have the authors made all data underlying the findings in their manuscript fully available (please refer to the Data Availability Statement at the start of the manuscript PDF file)?
The [PLOS Data policy](https://track.editorialmanager.com/CL0/https:%2F%2Fjournals.plos.org%2Fglobalpublichealth%2Fs%2Fdata-availability/1/010f019cad98b43b-a982877a-bc1a-4592-b4b0-a8f0b31cb2b3-000000/PoMaXSdbItYmdMPIy1IoVKEQ1u59ojqkfJhMxFKCjTw=252) requires authors to make all data underlying the findings described in their manuscript fully available without restriction, with rare exception. The data should be provided as part of the manuscript or its supporting information, or deposited to a public repository. For example, in addition to summary statistics, the data points behind means, medians and variance measures should be available. If there are restrictions on publicly sharing data—e.g. participant privacy or use of data from a third party—those must be specified. Reviewer #1: Yes; Reviewer #2: Yes; Reviewer #3: Yes

5. Is the manuscript presented in an intelligible fashion and written in standard English?
PLOS Global Public Health does not copyedit accepted manuscripts, so the language in submitted articles must be clear, correct, and unambiguous. Any typographical or grammatical errors should be corrected at revision, so please note any specific errors here. Reviewer #1: Yes: Reviewer #2: Yes: Reviewer #3: Yes

6. Review Comments to the Author
Please use the space provided to explain your answers to the questions above. You may also include additional comments for the author, including concerns about dual publication, research ethics, or publication ethics. (Please upload your review as an attachment if it exceeds 20,000 characters). Reviewer #1: good article; Reviewer #2: (No Response): Reviewer #3: Thank you for addressing reviewer comments from the previous round.

**Figure Resubmissions:**

While revising your submission, we strongly recommend that you use PLOS’s NAAS tool (<https://ngplosjournals.pagemajik.ai/artanalysis>) to test your figure files. NAAS can convert your figure files to the TIFF file type and meet basic requirements (such as print size, resolution), or provide you with a report on issues that do not meet our requirements and that NAAS cannot fix.

After uploading your figures to PLOS’s NAAS tool - <https://ngplosjournals.pagemajik.ai/artanalysis>, NAAS will process the files provided and display the results in the "Uploaded Files" section of the page as the processing is complete. If the uploaded figures meet our requirements (or NAAS is able to fix the files to meet our requirements), the figure will be marked as "fixed" above. If NAAS is unable to fix the files, a red "failed" label will appear above. When NAAS has confirmed that the figure files meet our requirements, please download the file via the download option, and include these NAAS processed figure files when submitting your revised manuscript.

**Response**

We thank the reviewers and editors for this helpful suggestion. All figures have been uploaded to the PLOS NAAS tool and processed in accordance with the journal’s requirements. The processed files have been incorporated into the revised manuscript, and all figures have been replaced with NAAS-compliant versions.

This article's conclusions and table discuss the need to decentralize PEP services to priority facilities.

I suggest the authors provide more specific criteria or models. For example: "In each ward with a population greater than X and/or located more than Y km from an existing PEP site, at least one medical facility (dispensary or health center) should be designated for rabies storage and vaccination. The selection of these facilities should be based on accessibility to the public (24/7) and the availability of trained personnel." This transforms a general recommendation into a measurable standard.

**Response:**
We thank the reviewer for this valuable suggestion. We have revised the manuscript to provide more specific and operational guidance on decentralizing PEP services. In the Conclusion and Recommendations section, we now include explicit criteria to guide implementation, suggesting that PEP providing facilities be designated in wards exceeding defined population thresholds and/or located beyond specified distances from existing PEP sites. We also outline key considerations for facility selection, including accessibility, staffing capacity, and infrastructure requirements for safe vaccine delivery. To ensure clarity and usability for policy and implementation, we have also updated Table 3 to include a concise, criteria-based recommendation aligned with this approach.
